# Characterising the dietary patterns of the European Roe Deer across biogeographical regions

**DOI:** 10.1007/s10344-025-02038-8

**Published:** 2025-12-16

**Authors:** Christopher Hirst, Robin Gill, Rob Ogden, Darren J. Shaw

**Affiliations:** 1 Forest Research, Northern Research Station, Midlothian, UK; 2 The Royal (Dick) School of Veterinary Studies and The Roslin Institute, The University of Edinburgh, Midlothian, UK; 3 Forest Research, Alice Holt Lodge, Wrecclesham, Surrey, UK

**Keywords:** Biogeographical regions, Herbivore diet, European ungulates

## Abstract

Biogeographical regions provide a framework for understanding species’ ecological patterns, yet their application to herbivore dietary studies remains limited. The European roe deer (*Capreolus capreolus*) presents an ideal candidate for biogeographical dietary analysis due to its widespread distribution and well-documented feeding patterns across Europe. Here, we analysed microhistological diet data using linear mixed-effects models to investigate regional and seasonal variations in roe deer feeding ecology across European Biogeographical Regions (EBRs), moving beyond traditional geographical classifications to understand dietary adaptations. The review encompassed 55 studies (54 included in statistical analyses) conducted over 52 years across 69 sites. While initially covering six EBRs, statistical analyses were constrained to three regions—Atlantic, Continental, and Mediterranean—due to insufficient data from the Boreal, Pannonian, and Alpine regions. We found complex dietary patterns, with intra-regional seasonal shifts in key food categories including coniferous trees, deciduous trees, shrubs, and half-woody plants. The Atlantic region showed significantly higher consumption of grasses, sedges, and rushes compared to Continental regions, while Mediterranean populations exhibited distinct autumn feeding patterns. Despite regional variations, consistent seasonal patterns in forb consumption were observed across all regions, peaking during spring and summer. Our findings suggest that roe deer modulate their feeding strategies according to regional conditions while maintaining some fundamental nutritional patterns. The biogeographical framework provides new understandings of herbivore dietary ecology not evident in previous studies using broader geographical classifications. These findings have important implications for understanding roe deer adaptation and informing regional management strategies.

**Supplementary Information:**

The online version contains supplementary material available at 10.1007/s10344-025-02038-8.

## Introduction

Biogeographical regionalisation is a fundamental concept in biogeography that categorises the world’s biota into meaningful geographic units that reflect ecological conditions and spatial patterns of biodiversity (Escalante 2009; Hengeveld 1992; Kreft and Jetz 2010; Mackey et al. 2008; Morrone 2018). The European Biogeographical Region (EBR) classification provides a framework for investigating spatial patterns in species distributions and ecology and is primarily delineated based on maps of European vegetation (see Cervellini et al. (2020) for full details). EBRs are widely applied in ecological studies, from assessing the impacts of climate change on terrestrial vertebrates (Maiorano et al. 2013) to spatial analysis of food webs (Galiana et al. 2021) and comparing plant phenology (Templ et al. 2017). Recent applications to dietary analysis have revealed important insights into spatial dietary patterns, trophic ecology and conservation management (Barnagaud et al. 2019; Díaz-Ruiz et al. 2013; Jacoby et al. 2023; Lanszki et al. 2016; Lozano et al. 2006; Romano et al. 2020), yet this approach remains unexplored for mammalian herbivores.

Large-scale spatial dietary patterns in herbivores, particularly ungulates, remain poorly characterised despite their extensive management and calls for consideration of their impact from local to large spatial scales (Gordon et al. 2004). This knowledge gap is surprising given herbivores’ reliance on plant communities, which strongly differ in composition and abundance across biogeographical regions and are central to defining these units (Cervellini et al. 2020; Roekaerts 2002). While biogeographical approaches have revealed dietary patterns in carnivores (Díaz-Ruiz et al. 2013; Lanszki et al. 2016; Lozano et al. 2006), similar analyses for herbivores are lacking.

Environmental heterogeneity drives ungulate distribution, habitat selection, and seasonal movements through its effects on food quality and availability (Mobæk et al. 2009; Mysterud et al. 2001; Van Beest et al. 2010). Investigating spatial changes in dietary habits is crucial for understanding local adaptation (Sanford et al. 2003), trophic roles and food web linkages (Lozano et al. 2006). It can reveal if species behave as trophic generalists or specialists across ecological gradients (Lozano et al. 2006). A biogeographical approach allows local findings to be ‘scaled-up’ (Hobbs 1996), identifying regional foraging patterns relevant to conservation and management.

Roe deer (*Caperolus capreolus*) represent an ideal species for biogeographical diet analysis as Europe’s most widespread ungulate. With an estimated population of 15 million (Apollonio et al. 2010; Lovari et al. 2016), roe deer inhabit a diversity of habitats (Linnell et al. 1998), spanning 7.2 million km^2^ from the Boreal forest of northern Europe to Mediterranean shrublands in the south (Burbaite and Csányi 2009). Although primarily associated with woodlands, their successful colonisation of diverse European landscapes, including forests, open countryside, areas of intensive agriculture, and peri-urban environments (Bresiński 1982; Linnell and Zachos 2011; McCarthy et al. 1996; Putman et al. 2014) is often attributed to their behavioural and digestive plasticity (Hewison et al. 2001; Jepsen and Topping 2004; Serrano Ferron et al. 2012). However, how this plasticity manifests in regional dietary differences remains poorly understood.

Roe deer diet varies with spatial heterogeneity in plant communities, climate and seasonality (Cornelis et al. 1999; Mysterud et al. 2001; Abbas et al. 2011), which likely leads to regional differences in diet. Previous reviews of microhistological dietary analysis studies in roe deer (Tixier and Duncan 1996; Cornelis et al. 1999; Spitzer et al. 2020) have attempted to capture this variation using different approaches. Studies have categorised habitats by dominant tree communities (e.g., deciduous, mixed, or coniferous woodlands) or agricultural status (Tixier and Duncan 1996; Cornelis et al. 1999; Spitzer et al. 2020), an approach that fails to capture the nuances in plant community compositions and their availability within these broadly defined habitat types, which can vary greatly within these broadly defined habitat types across the European continent (Cornelis et al. 1999).

At larger spatial scales, researchers have employed regional categorisations such as “northern”, “central”, and “southern” Europe (Spitzer et al. 2020), “Scandinavian”, “Continental”, and “Mediterranean” (Tixier and Duncan 1996), as well as gradients of altitude (Tixier and Duncan 1996), or longitude and latitude (Cornelis et al. 1999) to examine the impacts of region, environment, or climate on roe deer diet. Spitzer et al. (2020) found no significant difference in the proportion of browse and grasses in the diet of roe deer (and other ungulates) across broadly defined geographical regions, while Cornelis et al. (1999) detected effects of geographical location on diet, as measured by longitude and latitude, but no clear pattern could be determined. Tixier and Duncan (1996) observed variable diets across sites of similar altitudes or latitudes but could not identify consistent patterns.

These metrics inadequately represent differences in vegetation, phenology, and climate that influence dietary components across the roe deer’s range, limiting our ability to predict and manage their impacts as climate change alters plant communities. To address this, we utilise pre-defined biogeographical regions based on endemic flora and fauna communities (Cervellini et al. 2020; Roekaerts 2002; Templ et al. 2017), as a more biologically meaningful framework for characterising regional differences in the diet of a wide-spread herbivore.

Previous reviews have significantly advanced our understanding of seasonal and habitat effects on roe deer diet (Tixier and Duncan 1996; Cornelis et al. 1999; Spitzer et al. 2020). However, the two reviews that focused specifically on roe deer diet were conducted over 20 years ago (Tixier and Duncan 1996; Cornelis et al. 1999). Although Spitzer et al. (2020) provided a more recent review, it was part of a broader ungulate synthesis and did not focus deeply on roe deer diet and ecology, limiting the depth of insights specific to this species. Since the most recent roe deer-focused review in 1999, many additional local diet studies have been published across the species’ range. An updated synthesis of this larger body of literature, utilising the EBRs framework, could provide new understandings into regional variation in roe deer diet. This review is the first to utilise EBRs to investigate mammalian herbivore diet, analysing data from microhistological studies across biogeographical regions. We aimed to:

1. Characterise how the diet composition of European roe deer varies across EBRs.
2. Determine whether seasonal dietary patterns differ between biogeographical regions.
3. Identify any consistent dietary patterns across biogeographical regions that might indicate shared adaptations in roe deer foraging strategies.

## Materials and methods

### Selection criteria and methods for compiling studies on the diet of roe deer in Europe

To compile studies for this systematic review, we conducted a comprehensive literature search using the Boolean terms: “roe deer” AND diet* OR food* OR forage* (where “*” is a wildcard character). This search was conducted between January 2021 and March 2025 on Web of Science Core Collection, SCOPUS, and Google Scholar without restriction on publication year or language, except for papers in Russian due to translation limitations. Publications were initially filtered to identify studies employing quantitative methods for determining roe deer dietary composition. Reference lists from these studies were also examined to identify additional relevant sources (i.e., articles in non-indexed journals, PhD theses and MSc dissertations). Diet analysis using DNA metabarcoding approaches were not considered in this review as these methods may produce different dietary profiles compared to traditional microhistological analysis, potentially introducing methodological incompatibilities in our comparative framework.

A total of 76 studies were identified through our literature search and reference list examination. We applied a systematic filtering process with predefined exclusion criteria. First, we excluded studies relying solely on observational data (10 studies), such as bite counts and forage use, as direct feeding observations may not accurately identify specific plants selected from mixed vegetation, and estimating intake from observations is challenging due to difficulties distinguishing consumption from sampling behaviour (see Holechek et al. 1982). Additionally, converting observational data to mass or volume estimates requires conversion factors that introduce further uncertainty (Gross et al. 1993).

Further exclusions were made for studies that did not report full diet quantification or focused only on certain dietary components (5 studies), studies using feeding trials (2 studies), studies involving captive animals (2 studies), and studies only reporting annual diets not separated by season (2 studies).

These filtering steps ensured that the final selection of studies included only studies using microhistological analysis of faecal and/or rumen samples from wild or free-ranging roe deer populations in Europe. Following this selection process, 55 studies met all established criteria for inclusion in our descriptive analysis (see Supplementary Material). For statistical analysis, one study Marinucci (2005), was excluded as it did not report any of the six key food categories selected for quantitative comparison, resulting in 54 studies for statistical analysis (see Statistical Analysis section).

### Data extraction and standardisation

Dietary composition data were extracted from text, tables, and figures, using Web Plot Digitizer V.4.5 (automeris.io/WebPlotDigitizer/) to digitise data from graphs where necessary. All diet compositions were expressed in the same unit (i.e., the percentage of a diet component within the total diet). Following previous reviews (Cornelis et al. 1999; Spitzer et al. 2020), percentages of dietary components in the dry weight of rumen content, the volume of rumen content, the number of faecal fragments and the area of faecal fragments reported as a percentage of total fragments were all considered to represent a comparable proportion of dietary components.

Extracted data required transformation to compare across studies. Studies reporting trace amounts of dietary components were handled systematically; while reporting thresholds varied between publications (e.g., < 1%, < 0.01%), all components reported as trace amounts were excluded to prevent inflation bias of rare components. For dietary data extracted from graphs, proportions were adjusted to sum to 100% by multiplying all dietary components by the same factor when necessary, ensuring consistency with original reporting.

For studies reporting diet composition analyses for the same location but in separate seasons or reporting analyses from multiple sites, data were recorded separately and treated as different entries. Where multiple diets were reported for the same season and site, values were averaged to obtain a single representative diet prior to analysis. Time units were standardised by calculating seasonal means using consistent definitions: spring (March, April, and May), summer (June, July, and August), autumn (September, October, and November) and winter (December, January, and February). Sex was not included as a variable due to limited reporting, primarily only in rumen content studies.

**Fig. 1:**
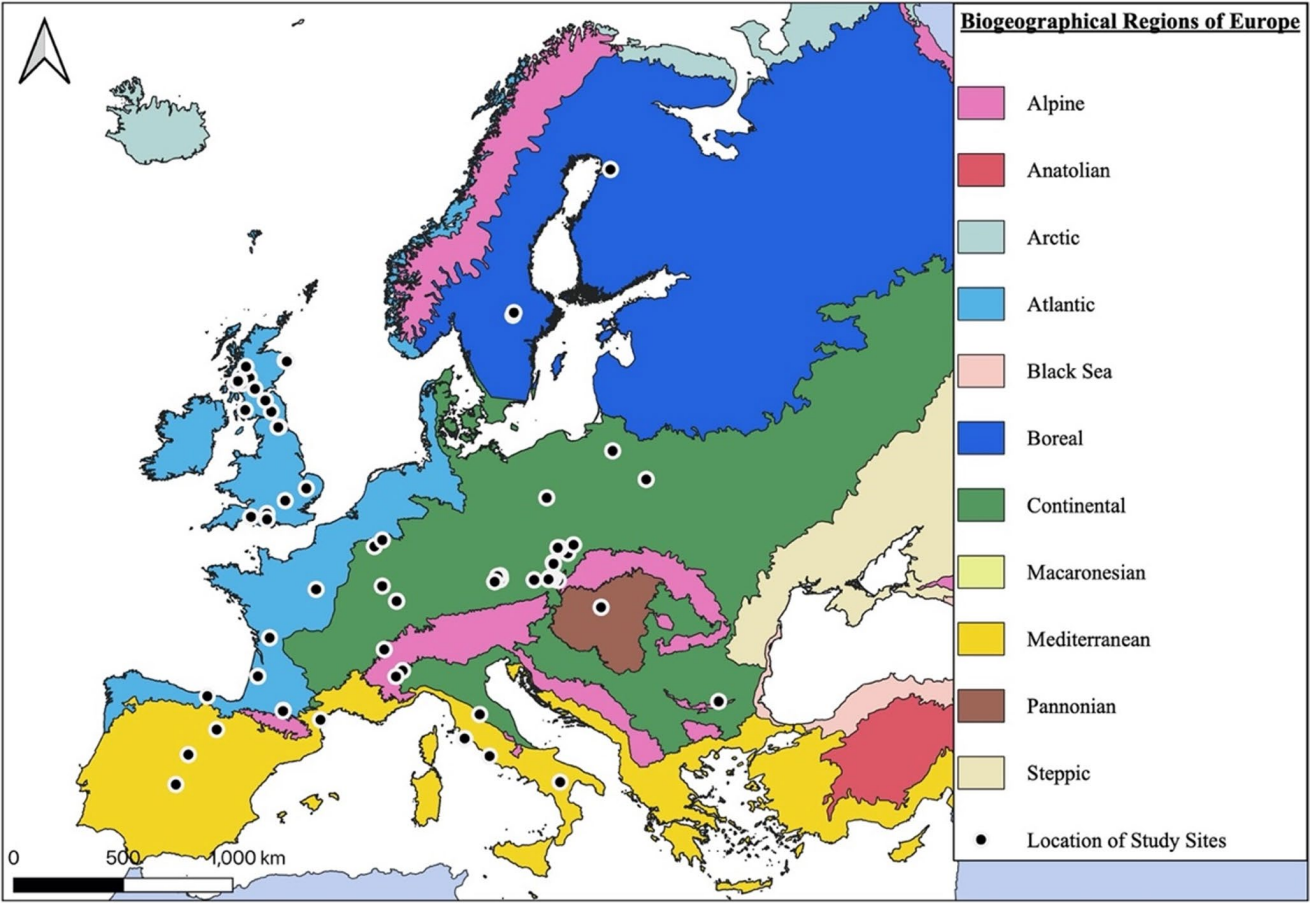
Map indicating European biogeographical regions (colours) and sites (black dots) included in this systematic review, prepared using QGIS (v.3.24.2). Map source: European Environment Agency (https://www.eea.europa.eu/data-and-maps/data/biogeographical-regions-europe-3)

Published geographic coordinates were used to categorise study sites into one of six European Environment Agency biogeographical regions: Alpine, Atlantic, Boreal, Continental, Mediterranean and Pannonian (Fig. 1).

Dietary components were classified into 16 food categories (Table 1) to allow comparison across studies reporting different taxonomical resolutions, however, not all of these were retained for statistical analysis (see Statistical Analysis). Unidentified fragments and coarse groupings (e.g., “woody browse”) were classified as “Unidentified” and “Uncategorised” respectively.

**Table 1:** Categories used to compare roe deer diet across 55 studies. The food categories are used for the descriptive analysis of diet, while the category for statistical analysis is used for subsequent analysis

Food Category	Category for Statistical Analysis	Description
Coniferous Trees	Coniferous Trees	Coniferous Trees
Deciduous Trees	Deciduous Trees	Deciduous Trees
Shrubs	Shrubs	Shrubs
Half -Woody Plants	Half -Woody	e.g., *Clematis*, *Lonicera*, *Hedera*,* Rosa*,* Rubus*
Forbs	Forbs	Herbaceous flowering plants that are not a graminoid
Ferns	Excluded from analysis	Ferns
Grasses, Sedges and Rushes (GSRs)	Grasses, Sedges and Rushes (GSRs)	Graminoids (*Poaceae*,* Cyperaceae* and *Juncaceae*)
Mosses	Excluded from analysis	Mosses
Fungi	Excluded from analysis	Fungi
Lichen	Excluded from analysis	Lichen
Cultivated Crops (Grasses)	Excluded from analysis	Agricultural graminoid plants
Other Cultivated Crops	Excluded from analysis	Non-graminoid agricultural
Non-plant food	Excluded from analysis	Vertebrates, invertebrates, and feed
Unidentified	Excluded from analysis	Fragments not identified in the original study
Uncategorised	Excluded from analysis	Fragments identified in the original study that were coarsely described and did not fit into the above categories
Other	Excluded from analysis	Items not fitting into the above categories, i.e., stonecrop species

### Statistical analysis

Of the 55 studies included in the descriptive review, 27 used faecal analysis, and 31 used rumen analysis (three studies employed both methods). Due to the limited number of studies available on roe deer diet, data were not separated by the analysis methods, reflecting the same approach as previous reviews (Cornelis et al. 1999; Spitzer et al. 2020). Instead, the source of dietary material was treated as a random effect in analyses to account for potential methodological biases. This allowed for a more comprehensive analysis of the available data.

The limited data from the Alpine (2 studies), Boreal (3) and Pannonian (6) biogeographical regions did not allow for robust statistical comparisons across all biogeographical regions. Therefore, analysis was restricted to studies from the Atlantic, Continental, and Mediterranean regions where more data were available to make reliable inferences about regional differences in diet. Six food categories - coniferous trees, deciduous trees, forbs, grasses sedged and rushes (GSRs), shrubs, and half-woody plants - were selected for further analysis as they were frequently reported and present across the subset of biogeographical regions included in the analysis (Table 1).

A log-ratio transformation was applied to make the data more amenable to statistical analyses by preserving the relevant information contained in the ratios between dietary components and addressing the sum constraint inherent in compositional data (Corrêa Leite 2021). Specifically, an isometric log-ratio (ILR) transformation was used, which involves breaking down the proportions of dietary components into non-overlapping groups and representing the relationship between these groups as log-ratios (Egozcue et al. 2003).

To perform the ILR transformation, the geometric mean of each diet was first calculated by multiplying the proportions of all sixteen food categories present (> 0) in a reported diet and then taking the *n*^*th*^ root, where *n* represents the number of food categories with proportions greater than zero in that specific diet. If all food categories had proportions greater than zero, *n* would be equal to sixteen. However, if any of the six food categories being used for further analysis had a proportion of zero, *n* would be one less for each such category. Subsequently, the ILR transformation was applied by dividing the diet proportions by the geometric mean. Where zeros were present, they were converted to NA values and omitted from the analysis. This allowed a focused analysis of only the categories present in roe deer diets according to the studies reviewed. One study by Marinucci 2005) was excluded from statistical analysis, as none of the chosen six food categories were present in the reported diet. In total, 54 of the 55 reviewed studies were retained for statistical analysis.

Linear mixed-effects models were constructed for each of the six food categories using the lmer function from the *lme4* R package (Bates et al. 2015). Models included fixed effects of season, biogeographical region, and their interaction. Random effects included study ID (ref) to account for the non-independence of data collected from multiple study sites within the same study, and for potential biases associated with different dietary sources (i.e., rumen content or faeces).

The incorporation of diet source as a random effect in models aims to address the potential source of variability in diet reconstructions that can occur due to the choice of dietary material. While some studies have found similar reconstructions of diet using both faecal and stomach content analysis (Homolka and Heroldová 1992; Robert and Norman 1974), others have reported significant differences (Cornelis et al. 1999). These discrepancies mainly arise from the differential digestibility of food items and tissue types (Leslie et al. 1983; Vavra and Holechek 1980), and that the rate of digestibility may differ between faeces and stomach content. Rumen contents, being less digested, may better preserve softer plant tissues (Johnson et al. 1983; McInnis et al. 1983), while faecal samples might disproportionately represent more resilient plant fragments (Leslie et al. 1983; Stewart 1967).

The initial model selection aimed to assess the dependency of the log-ratio of each of the six food categories on the interaction between season (spring, summer, autumn, and winter) and biogeographical region (Atlantic, Continental, and Mediterranean). Several models were tested using the lmer function, and the models were compared using the log-likelihood ratio test (LRT) statistics obtained through the lrtest function in the *lmtest* R package (Hothorn 2002).

- Model 1: log-ratio ~ season * biogeographical region + (ref) + (dietary source).
- Model 2: log-ratio ~ season + biogeographical region + (ref) + (dietary source).

If Model 2 was determined to have the best fit, the significance of the fixed effects season and biogeographical region were tested using Tukey’s post hoc tests to evaluate pairwise differences. Non-significant fixed effect terms were then dropped from Model 2.

To examine interactions between season and biogeographical regions where Model 1 was found to be the best model, additional linear mixed-effects models were run within season and region subgroups (Models 3 and 4). Tukey’s post hoc tests were then used to evaluate pairwise differences.

- Model 3: log-ratio ~ season + (ref) + (dietary source), subset = biogeographical region = “x”.
- Model 4: log-ratio ~ biogeographical region + (ref) + (dietary source), subset = season= “x”.

## Results

### Overview of studies and taxonomic resolution of diets

This review initially encompassed 55 studies on roe deer diet, spanning 52 years (1969 to 2021) and covering 69 study sites across six biogeographical regions in Europe (See Table 2). The Atlantic, Continental and Mediterranean regions were the most extensively studied, with 29, 20 and nine sites, respectively. In contrast, the Alpine, Boreal, and Pannonian regions had limited representation, with only two to six sites each. Diet composition was assessed via rumen analysis in 31 studies and faecal analysis in 27 studies, with three studies employing both methods (Hosey 1974, Henry 1975, Johnson 1984).

**Table 2:** Overview of the 55 roe deer diet studies by biogeographical region, method, and season. Numbers in brackets refer to the number of diets reviewed, in which a diet represents the data from each season and site for each study

BGR	Sites	Studies	Diet Analysis Method		Number of Studies per Season	
Alpine	2	2 (*5*)	Rumen: Faecal:	0 (*0*) 2 (*5*)	Spring: Summer: Autumn: Winter:	1 (*1*) 2 (*2*) 1 (*1*) 1 (*1*)
Atlantic	29	22 (*111*)	Rumen: Faecal:	15 (*67*) 10 (*44*)	Spring: Summer: Autumn: Winter:	12 (*15*) 17 (*26*) 15 (*23*) 22 (*47*)
Boreal	3	3 (*8*)	Rumen: Faecal:	2 (*6*) 1 (*2*)	Spring: Summer: Autumn: Winter:	1 (*1*) 2 (*2*) 1 (*1*) 3 (*4*)
Continental	20	16 (*68*)	Rumen: Faecal:	10 (*25*) 6 (*43*)	Spring: Summer: Autumn: Winter:	10 (*15*) 11 (*17*) 12 (*17*) 14 (*19*)
Mediterranean	9	7 (*36*)	Rumen: Faecal:	3 (*14*) 4 (*22*)	Spring: Summer: Autumn: Winter:	3 (*9*) 3 (*10*) 5 (*7*) 5 (*10*)
Pannonian	6	6 (*17*)	Rumen: Faecal:	2 (*2*) 4 (*15*)	Spring: Summer: Autumn: Winter:	4 (*4*) 3 (*3*) 3 (*3*) 6 (*7*)
Total	**69**	**55 (*265*)**	Rumen: Faecal: Both	**31 (*114*)** **27 (*131*)** **3**	Spring: Summer: Autumn: Winter:	**30 (*45*)** **29 (*60*)** **34 (*52*)** **50 (*88*)**

The 55 studies reviewed reported a total of 703 dietary components, 101 of which were reported only in trace amounts. Of the dietary components reported, 336 were identified at the species level and 108 at the genus level, demonstrating the diverse diet of roe deer across Europe. The remaining were listed in coarser taxonomic groupings. The studies reported consumption of thirteen coniferous tree species, seven cultivated crop species, four grass-like cultivated crop species, 62 deciduous tree species, nine fern species, 135 forb species, 43 GSR species, 19 half-woody plant species, five lichen species, four moss species and 35 shrub species. The most common taxa are shown in Table 3.

**Table 3:** Top 10 dietary taxa consumed by roe deer across 55 studies within their European range are ranked by mean proportion and frequency of occurrence (FOO). Mean (%) shows the average proportion of each taxon across 265 diets. Values in parentheses show the maximum percentage observed (the minimum was 0% for all taxa). FOO (%) shows the percentage of diets (out of 265) in which the taxon was reported. Values in parentheses show the number of diets. A diet represents data from each season and site for each study. For genera, percentages include the combined value for components reported at the genus level and species conglomerated into respective genera. Species are ranked separately from genera. The food category column shows how each taxon is grouped for analysis

Rank	Species	Food Category	Mean (%)	Genera	Food Category	Mean (%)
1	*Calluna vulgaris*	Shrub	6 (71)	*Rubus*	Half-Woody	13 (98)
2	*Hedera helix*	Half-Woody	2 (55)	*Calluna*	Shrub	6 (71)
3	*Rubus fruitcosus*	Half-Woody	2 (78)	*Quercus*	Deciduous Tree	5 (87)
4	*Vaccinium myrtillius*	Shrub	1 (29)	*Vaccinium*	Shrub	2 (31)
5	*Rubus ulmifolius*	Half-Woody	1 (98)	*Hedera*	Half-Woody	2 (55)
6	*Pinus sylvestris*	Coniferous Tree	1 (32)	*Picea*	Coniferous Tree	2 (31)
7	*Zea mays*	Cultivated Crop (Grasses)	1 (37)	*Pinus*	Coniferous Tree	1 (32)
8	*Picea abies*	Coniferous Tree	1 (25)	*Zea*	Cultivated Crop (Grasses)	1 (37)
9	*Medicago sativa*	Forb	1 (33)	*Medicago*	Forb	1 (33)
10	*Picea sitchensis*	Coniferous Tree	1 (18)	*Dryopteris*	Fern	1 (24)
**Rank**	**Species**	**Food Category**	**FOO (%)**	**Genera**	**Food Category**	**FOO (%)**
1	*Calluna vulgaris*	Shrub	26 (68)	*Rubus*	Half-Woody	49 (130)
2	*Vaccinium myrtillius*	Shrub	20 (53)	*Vaccinium*	Shrub	31 (83)
3	*Hedera helix*	Half-Woody	17 (46)	*Quercus*	Deciduous Tree	31 (83)
4	*Picea abies*	Coniferous Tree	15 (39)	*Calluna*	Shrub	26 (68)
5	*Pinus sylvestris*	Coniferous Tree	14 (37)	*Picea*	Coniferous Tree	25 (66)
6	*Fagus sylvatica*	Deciduous Tree	9 (24)	*Hedera*	Half-Woody	22 (57)
7	*Picea sitchensis*	Coniferous Tree	8 (22)	*Pinus*	Coniferous Tree	19 (49)
8	*Ligustrum vulgare*	Shrub	8 (20)	*Trifolium*	Forb	14 (36)
9	*Corylus avellana*	Deciduous Tree	7 (19)	*Rosa*	Half-Woody	13 (34)
10	*Dactylis glomerata*	GSRs	7 (18)	*Rumex*	Forb	12 (32)
				*Betula*	Deciduous Tree	12 (32)

### Descriptive analysis of roe deer diet

**Fig. 2:**
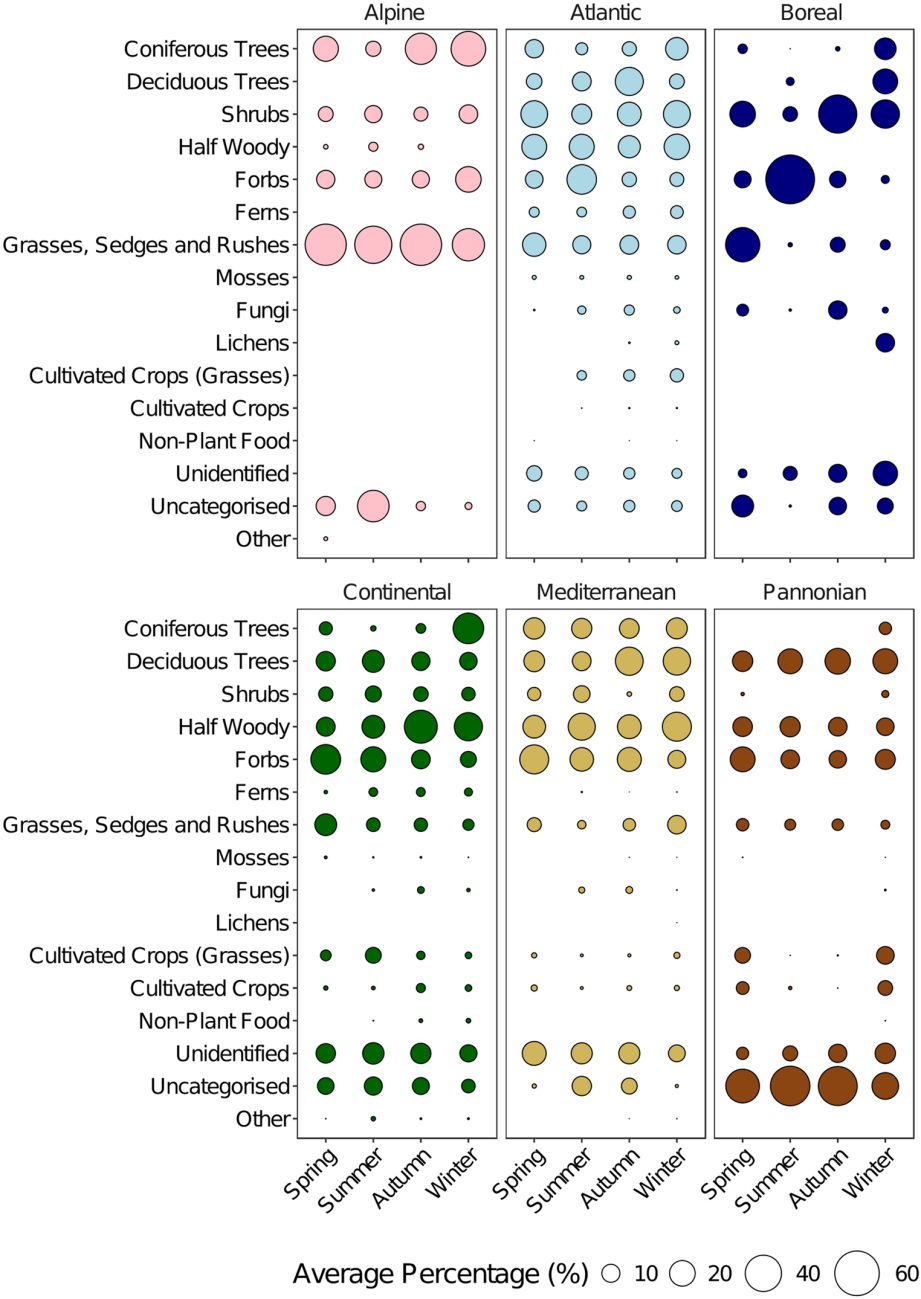
The mean percentage of sixteen food categories that were identified in the 55 studies of roe deer diet categorised by both biogeographical region and season

Descriptive analysis of dietary composition from 55 studies across biogeographical regions revealed distinct patterns in roe deer diet (Fig. 2). The Atlantic, Continental, and Mediterranean regions, which were better studied, exhibited a higher richness of food categories, likely providing a more comprehensive representation of roe deer diet. Across all six biogeographical regions, coniferous trees, deciduous trees, shrubs, half-woody plants, forbs and GSRs constituted substantial proportions of the diet.

Atlantic diets showed the highest diversity, encompassing 15 food categories, with notable proportions of half-woody plants, shrubs, and coniferous and deciduous trees. Similarly, Continental diets reported 15 food categories, displaying high mean proportions of half-woody plants, forbs, and deciduous trees. Mediterranean diets, reporting 14 food categories, were characterised by the dominance of coniferous trees, deciduous trees, half-woody plants, and forbs.

In contrast, the less-studied regions had less-rich diets. Alpine diets, reporting only seven food categories, were dominated by GSRs and coniferous trees. Boreal diets, encompassing nine food categories, showed high mean proportions of shrubs across seasons and forbs in summer. Pannonian diets, reporting 13 food categories, were notable for a high proportion of uncategorised items, along with substantial contributions from deciduous trees, half-woody plants, and forbs.

These regional variations in diet composition and diversity highlight the adaptability of roe deer feeding strategies across different European landscapes. However, the disparities in sampling effort between regions should be considered when interpreting these patterns, particularly for the less-studied Alpine, Boreal, and Pannonian regions.

### Regional variation in roe deer diet composition

The statistical analysis of 54 studies focused on six key food categories: coniferous trees, deciduous trees, forbs, GSRs, shrubs and half-woody plants. These categories comprised on average 76.09% ± 1.58% of the overall diet, ranging from 3.5% to 100%.

**Fig. 3:**
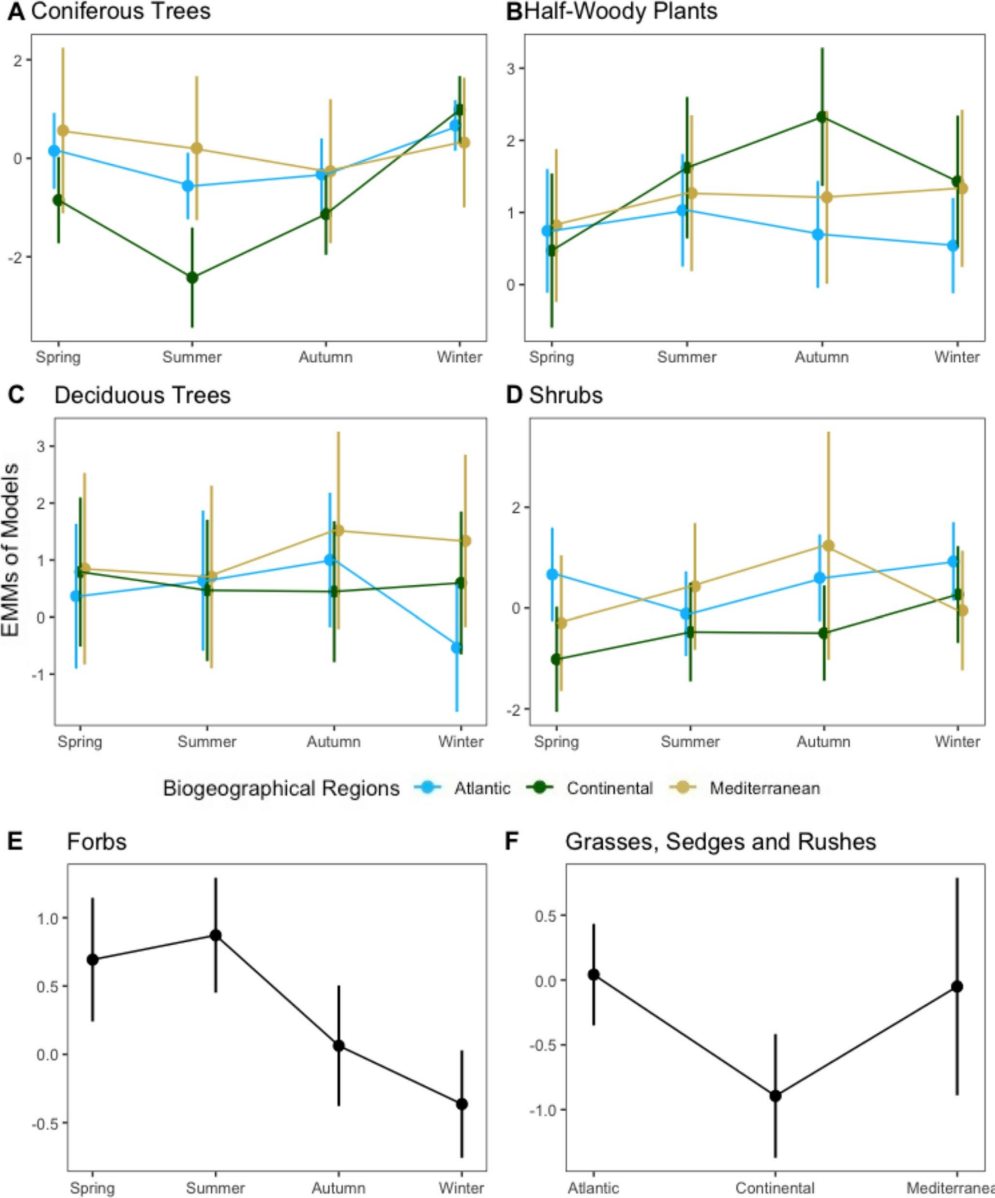
Estimated marginal means and standard error from linear mixed effect models explaining the variation in the isometric log-ratio (ILR) transformed compositional data for six food categories consumed by roe deer. The ILRs of **A**) coniferous trees, **B**) half-woody plants, **C**) deciduous trees and **D**) shrubs were best explained by the interaction between season and biogeographical region. The ILRs of **E**) forbs were best explained by season, and **F**) grasses sedges and rushes (GSRs) by biogeographical region

Linear mixed models revealed significant regional variations in diet composition. Simplified models best explained the variation in the proportions of GSRs (Model 4) (Fig. 3F), with Continental diets containing significantly lower proportions of GSRs than Atlantic diets (39% lower: Est=−0.94, SE = 0.31, *p* = 0.008). Regional differences were also evident for other food categories, but only when considering seasonal interactions.

In winter, the proportions of deciduous trees were significantly higher in the Continental (3.8 times higher: Est = 1.34, SE = 0.47, *p* = 0.012) and Mediterranean (5.4 times higher: Est = 1.68, SE = 0.63, *p* = 0.021) than in the Atlantic biogeographical region. Additionally, spring diets from the Continental biogeographical regions had significantly lower proportions of shrubs than in the Atlantic (77% lower: Est=−1.49, SE = 0.56, *p* = 0.021).

## Seasonal patterns in diet composition across biogeographical regions

Model selection indicated that the interaction between season and biogeographical region best explained the variation in the proportion of coniferous trees, half-woody plants, deciduous trees and shrubs in diets (Fig. 3A - D).

For coniferous trees, Atlantic diets showed lower proportions in autumn (62% lower: Est=−0.98, SE = 0.36, *p* = 0.032) and summer (70% lower: Est=−1.22, SE = 0.33, *p* = 0.001) diets than in winter. In Continental regions, winter diets had higher proportions of coniferous trees compared to autumn (88% lower: Est=−2.12, SE = 0.46, *p* < 0.001), spring (84% lower: Est=−1.83, SE = 0.48, *p* = 0.001), and summer (97% lower: Est=−3.41, SE = 0.55, *p* < 0.001), and lower proportions in summer diets than spring (79% lower: Est=−1.57, SE = 0.58, *p* = 0.033).

Deciduous trees in Atlantic diets had higher proportions in autumn (4.5 times higher: Est = 1.51, SE = 0.40, *p* = 0.001) and summer (3.3 times higher: Est = 1.20, SE = 0.46, *p* = 0.041) than in winter. Whereas, in the Mediterranean biogeographical region, spring (61% lower: Est=−0.95, SE = 0.35, *p* = 0.036) and summer (64% lower: Est=−1.01, SE = 0.34, *p* = 0.012) diets had lower proportions of deciduous trees than autumn.

Half-woody plants in Continental diets exhibited lower proportions in spring than in autumn (87% lower: Est=−2.03, SE = 0.63, *p* = 0.007). Proportions of shrubs in Atlantic diets were lower in summer compared to winter (64% lower: Est=−1.03, SE = 0.26, *p* < 0.001), while in the Continental region, they were lower in spring than in winter (72% lower: Est=−1.27, SE = 0.43, *p* = 0.015).

### Consistent seasonal dietary patterns across biogeographical regions

While many dietary components showed intra-regional seasonal patterns, forbs exhibited a consistent seasonal trend across all regions (Model 3) (Fig. 3E). Proportions of forbs were significantly higher in summer than in winter (3.5 times higher: Est = 1.24, SE = 0.19, *p* < 0.001) and autumn (2.3 times higher: Est = 0.81, SE = 0.21, *p* < 0.001), and higher in spring than in winter (2.9 times higher: Est = 1.06, SE = 0.21, *p* < 0.001) and autumn (1.9 times higher: Est = 0.63, SE = 0.23, *p* = 0.028).

## Discussion

Our biogeographical analysis of the European roe deer diet, synthesising 55 studies (54 included in statistical analysis) across 69 sites, six biogeographical regions and 52 years, revealed complex patterns of regional dietary variation undetected in previous reviews (Tixier and Duncan 1996; Cornelis et al. 1999; Spitzer et al. 2020). The Atlantic, Continental, and Mediterranean regions exhibited a higher dietary richness of food categories than the Boreal, Alpine and Pannonian biogeographical regions. This disparity in research efforts across biogeographical regions both limits our current understanding and highlights opportunities for future research to complete the picture of roe deer dietary variation across Europe. However, statistical analysis of the Atlantic, Continental, and Mediterranean regions demonstrated both independent and interactive effects of season and region on key food categories, highlighting the species’ dietary flexibility. Rather than exhibiting fixed inter-regional patterns, roe deer showed adaptable feeding strategies linked to local plant phenology and food availability. Through this biogeographical approach, we identified how these adaptations manifest in specific regional contexts, providing important insights into the ecological mechanisms underlying diet selection. This review illustrates how a macroecological perspective can reveal detailed dietary patterns that demonstrate the ways generalist herbivores modulate their nutritional strategies across diverse landscapes and seasons.

### A descriptive review of roe deer diet

Our findings confirm roe deer as generalist mixed feeders, consuming a diverse array of plant, fungi, and lichen taxa, with 336 species recorded in their diets − 31 more than previously documented (Tixier and Duncan 1996), and over twice that reported for European red deer (145 species; Gebert and Verheyden-Tixier 2001). Key dietary items included *Calluna vulgaris*, *Vaccinium myrtillus*,* Hedera helix*, and the genera *Rubus*,* Quercus*,* Picea*, and *Pinus*, while the presence of agricultural crops like *Zea mays* highlighted adaptation to human-modified landscapes.

Our characterisation of roe deer as generalist mixed feeders consuming diverse plant taxa, including significant proportions of graminoids and forbs, contributes to a long-standing debate about herbivore feeding classifications. Building on traditional grazer-browser distinctions, Hofmann and Stewart (1972) developed a more detailed three-category system: concentrate selectors (browsers selecting diets containing at least 75% fruits, dicot foliage, and woody browse), intermediate feeders (selecting both grass and browse), and grass and roughage feeders (selecting diets containing less than 25% browse). Hofmann (1989) further refined this framework, positioning roe deer as the archetypal concentrate selectors—characterised by their preference for high-quality diets rich in plant cell contents but low in fibre and adapted for processing easily digestible forage while avoiding fibrous roughage.

However, this rigid classification faced criticism as the anatomical and physiological foundations of Hofmann’s work were further tested. Gordon and Illius (1994) demonstrated that after controlling for body mass, digestive parameters showed little difference between supposed browsers and grazers, while Robbins et al. (1995) concluded that many of Hofmann’s physiological interpretations were not supportable, noting that dietary differences reflected the chemical and physical characteristics of available food rather than fundamental differences in ruminant anatomy. These critiques highlighted that anatomical features did not predict feeding behaviour as strictly as originally proposed.

Contemporary research has moved toward recognising the flexible nature of herbivore feeding strategies. Gordon and Prins (2019) noted that both selective and unselective species exist within traditional grazer and browser categories, while König et al. (2020) found roe deer consuming crude fibre at levels comparable to species classified as grazers or intermediate feeders—leading them to propose replacing “concentrate selector” with simply “selector” to avoid misinterpretations.

Our biogeographical findings support this move toward recognising feeding flexibility. The significant regional consumption of graminoids, the consistent cross-regional pattern of forb utilisation, and the documentation of a large number of plant species in European roe deer diets demonstrate feeding flexibility that transcends the rigid browser and concentrate selector classification, supporting the more flexible ‘selector’ concept. This biogeographical evidence suggests that roe deer could be thought of as generalist mixed feeders, reflecting their consumption of diverse plant taxa across multiple functional groups depending on phenological and spatial availability rather than specialisation on high-quality browse alone.

### Biogeographical trends

GSRs comprised a significantly higher proportion of the diets of roe deer in the Atlantic compared to the Continental region, likely reflecting differences in habitat composition between biogeographical regions. The Atlantic region contains over twice the grassland cover but half the forest cover of the Continental region (Condé et al. 2002a, b), demonstrating how the habitat composition of regions may influence feeding strategies along the grazing-browsing continuum.

Most other dietary components exhibited more complex patterns of region-season interactions. Winter consumption of deciduous trees was higher in Continental and Mediterranean regions compared to the Atlantic region, possibly reflecting differences in winter foraging strategies adapted to local vegetation composition and phenology. In Continental and Mediterranean regions, deciduous trees might retain leaves longer or provide more accessible browse (e.g., twigs, buds) during winter compared to the Atlantic region (Preislerová et al. 2024). Additionally, the greater forest cover in Continental regions (Condé et al. 2002a, b), likely increases winter deciduous browse availability when other food sources are scarce. Conversely, Continental spring diets contained fewer shrubs than the Atlantic region, suggesting earlier availability of preferred forbs or regional differences in shrub phenology (Templ et al. 2017). The prevalence of interaction effects, rather than independent regional effects, suggests seasonal shifts within biogeographical regions more strongly influence diet than universal seasonal patterns.

### Interactions between season and biogeographical regions

Atlantic and Continental roe deer increase their winter intake of woody browse from conifers and shrubs, likely driven by reduced forage selectivity during resource scarcity (Tixier et al. 1997). Despite conifers’ low palatability and negative effects on fitness due to secondary compounds (Oh et al. 1967; Duncan et al. 1994; van Genderen et al. 1996; de Jong et al. 2004), in the absence of alternative forage, they serve as fallback food when locally abundant (Barančeková et al. 2010; Verheyden et al. 1998). This pattern of winter conifer browsing is documented across countries within the Continental and Atlantic regions of Europe, such as Scotland and France (Latham et al. 1999; Maizeret and Ballon 1990; Welch et al. 1991), but also in Sweden and Finland (Bergquist and Örlander 1996; Helle 1980), and is likely exacerbated in conifer plantations where coniferous crops are prioritised over other alternative vegetation. The concurrent increase in winter shrub consumption in the Continental and Atlantic supports the idea that increasing alternative winter forage shrubs such as *Calluna vulgaris* and *Vaccinium myrtillus* could reduce browsing pressure on commercial conifers (Ward et al. 2008; Welch et al. 1991). Winter emerged as the season most frequently showing significant differences in dietary composition across regions, highlighting its critical role in driving localised dietary shifts.

Atlantic populations increased deciduous browse in summer and autumn (compared to winter), coinciding with the seasonal peak in nutrient availability of deciduous leaves and fruits, which are a key resource for roe deer (González-Hernández and Silva-Pando 1999; Moser et al. 2006). In contrast, Continental populations favoured half-woody plants in autumn, suggesting localised preferences that could be driven by differences in habitat composition and vegetation communities (Condé et al. 2002a, b). The Continental region demonstrated the most pronounced seasonal dietary variability, with significant shifts observed across four major food categories throughout the year, while the Atlantic region showed slightly less extensive seasonal variation.

Mediterranean populations showed distinct seasonal patterns in deciduous tree consumption, with significantly higher proportions in autumn compared to spring and summer. This pattern reflects the unique phenology of Mediterranean ecosystems, where hot, dry summers and mild, dry winters are interspersed with wet spring and autumn seasons, creating distinct seasonal growing patterns. Mediterranean summers impose drought conditions that limit plant growth and lead to nutritional constraints for herbivores, similar to winters in Northern Europe (Bugalho and Milne 2003; Pignatti 1994). As forb-rich field layers in Mediterranean forests and wooded habitats dry out due to increasing soil water deficits (Mooney and Kummerow 1981), deciduous trees become one of the few remaining sources of nutritious forage leading into autumn. The subsequent autumn rains trigger new leaf growth in many Mediterranean plants (Larcher 2000). This autumn leaf flush may provide an abundance of palatable new leaves and fruits from deciduous trees for roe deer, some of which will persist into the winter months (Cavender-Bares et al. 2005; Larcher and Bauer 1981; Minder 2012). Overall, the Mediterranean region exhibited greater dietary stability than the Continental and Atlantic regions, with seasonal changes primarily limited to deciduous tree consumption, possibly reflecting less pronounced seasonal fluctuations in food availability.

These regional variations in seasonal diet demonstrate adaptive foraging responses to local habitat composition, plant phenology, and climate. The contrasts between growing and non-growing seasons highlight how roe deer vary their diets with seasonal vegetation changes, while the differing degrees of dietary flexibility across biogeographical regions suggest roe deer modulate their feeding strategies most dynamically, where seasonal resource fluctuations are greatest. This analysis reveals patterns not evident in previous local studies and emphasises the importance of macroecological perspectives in understanding herbivore impacts across their range.

### Overarching seasonal trends

While many dietary components showed intra-regional seasonal patterns, forb consumption displayed a consistent seasonal trend across all biogeographical regions. Forbs constituted significantly higher proportions of roe deer diet in spring and summer compared to autumn and winter. This pattern, documented across multiple site-level studies (Barančeková 2004; Barančeková et al. 2010; Gębczyńska 1980; Jackson 1980; Latham et al. 1999), reflects the peak productivity and nutritional quality for forbs throughout much of Europe, which are highly abundant in spring and early summer. The consistency of this pattern suggests a fundamental nutritional strategy that transcends regional differences in habitat and climate, with roe deer selectively targeting this high-quality, seasonally available forage.

It is important to note that this pattern persisted even in Mediterranean regions, despite expectations that hot, dry summers would lead to earlier forb senescence (Mooney and Kummerow 1981). This unexpected result might reflect significant ecological variation within the Mediterranean biome, with study sites potentially representing higher altitudes, moderate coastal areas (Freschi et al. 2021; Minder 2012), or localities with reliable summer precipitation.

### Efficacy of the biogeographical approach

The EBR framework provides enhanced macroecological insights compared to previous approaches that use broadly defined habitats or geographical boundaries (Tixier and Duncan 1996; Cornelis et al. 1999; Spitzer et al. 2020). By categorising Europe into units that reflect endemic flora and environmental conditions, this method offers more ecologically meaningful distinctions, revealing dietary patterns linked directly to regional biotic and abiotic contexts that shape food resource availability.

Similar biogeographical approaches have proven valuable across diverse taxa, yielding insights into spatial dietary patterns, trophic ecology, and conservation management (Barnagaud et al. 2019; Díaz-Ruiz et al. 2013; Jacoby et al. 2023; Lanszki et al. 2016; Lozano et al. 2006; Romano et al. 2020), as well as climate change impacts on vertebrates (Maiorano et al. 2013), and plant phenology (Templ et al. 2017).

The biogeographical approach utilised in this review represents an advancement in understanding roe deer dietary ecology, revealing patterns that were obscured in previous broad-scale or localised studies. By integrating both spatial and temporal factors within an ecologically meaningful framework, our analysis demonstrates the importance of biogeographical context in herbivore diet studies. Understanding regional and seasonal dietary patterns can inform habitat management strategies to address browsing impacts in different biogeographical contexts. Future research could examine how climate change might alter these dietary patterns and extend similar frameworks to other widespread herbivores to gain comparative insights into feeding adaptability across diverse landscapes.

## Supplementary Information

Below is the link to the electronic supplementary material.

Supplementary Material 1 (XLSX 20.4 KB)

## Data Availability

The dataset supporting the conclusions of this article is available from the corresponding author on reasonable request.
